# Visual Control of Robots Using Range Images

**DOI:** 10.3390/s100807303

**Published:** 2010-08-04

**Authors:** Jorge Pomares, Pablo Gil, Fernando Torres

**Affiliations:** Physics, Systems Engineering and Signal Theory Department, University of Alicante, PO Box 99, Alicante 03080, Spain; E-Mails: Pablo.Gil@ua.es (P.G.); Fernando.Torres@ua.es (F.T.)

**Keywords:** visual servoing, ToF cameras, self-calibration, robotics

## Abstract

In the last years, 3D-vision systems based on the time-of-flight (ToF) principle have gained more importance in order to obtain 3D information from the workspace. In this paper, an analysis of the use of 3D ToF cameras to guide a robot arm is performed. To do so, an adaptive method to simultaneous visual servo control and camera calibration is presented. Using this method a robot arm is guided by using range information obtained from a ToF camera. Furthermore, the self-calibration method obtains the adequate integration time to be used by the range camera in order to precisely determine the depth information.

## 1. Introduction

Nowadays, visual servoing is a well known approach to guide a robot using visual information. The two main types of visual servoing techniques are position-based and image-based [1]. The first one uses 3-D visually-derived information when making motion control decisions. The second one performs the task by using information obtained directly from the image. However, the interaction matrix employed in these visual servoing systems requires known different camera parameters and the depth of the image features.

A typical approach to determine the depth of a target is the use of multiple cameras. The most commonly applied configuration using more than one camera is stereo vision (SV). In this case, in order to be able to calculate the depth of a feature point by triangulation, the correspondence of this point in both cameras must be assured.

In this paper the use of 3D time-of-flight (ToF) cameras is proposed in order to obtain the required 3D information in visual servoing approaches. These cameras provide range images which give depth measurements of the visual features. In the last years 3D-vision systems based on the ToF principle have gained more importance compared to SV. Using a ToF camera, illumination and observation directions can be collinear, therefore, this technique does not produce incomplete range data due to shadow effects. Furthermore, SV systems have difficulties in estimating the 3D information of planes such as walls or roadways. They cannot find the corresponding physical point of the observed 3D-space in both camera systems. Hence the 3D information of that point cannot be calculated by applying the triangulation principle. Another standard technique to obtain 3D information is the use of laser scanners. The advantages of ToF cameras over laser scanners are the high frame rates and the compactness of the sensor. These aspects have motivated the use of a ToF camera to obtain the required 3D information to guide the robot.

Some previous works have been developed in order to guide a robot by visual servoing using ToF Cameras. Within these works, a visual servoing system using PSD (Position Sensitive Device) triangulation for PCB manufacturing is presented in [2]. In [3] a position-based visual servoing is described to perform the tracking of a moving sphere using a pan-tilt unit. In this last paper a ToF Camera manufactured by CSEM is used. A similar approach is described in [4] to determine object positions by means of an eye-to-hand camera system. Unlike these previous approaches, in this paper the range images are not used directly to estimate the 3D pose of the objects in the workspace. A new image-based visual servoing system which integrates range information in the interaction matrix is presented to perform the robot guidance. Another advantage of the proposed system over the previous ones is the possibility of performing the camera calibration during the task. To do so, the visual servoing system uses the range images not only to determine the depths of the features but also to adjust the ToF camera parameters during the task.

When a ToF camera is used, some aspects must be taken into consideration, such as large fluctuations in precision caused by external interfering factors (e.g., sunlight) and scene configurations (*i.e.*, distances, orientations and reflectivity). These influences produce systematic errors which must be processed. Specifically, the distance computed from the range images is very changing depending on the integration time parameter. This paper presents a method for the online adaptation of the integration time of ToF cameras. This online adaptation is necessary to capture the images in the best condition independently of the changes in distance (between camera and objects) caused by the movements of the camera when it is mounted on a robotic arm. Previous works have been developed for ToF camera calibration [5–7]. These works perform an estimation of the camera parameters and distance errors when static scenes are observed. In these researches, a fixed distance between the camera and the objects is considered. Therefore, these previous works cannot be applied in visual servoing tasks where the camera performs the tracking of a given trajectory. In this last case, the camera parameters such as the integration time must be modified in order to optimally observe the scene. To do this, several previous works adapt the camera parameters, such as the amplitude of the integration time, during the task. In [8] a CSEM-Swissrange camera is employed for the navigation of a mobile robot in an environment with different objects. This work automatically estimates the value of the integration time according to the intensity pattern obtained by the camera. However, this parameter is depends on illumination and reflectance conditions. To solve this problem, in [9] a PMD camera is also used for mobile robot navigation. This work proposes an algorithm based on the amplitude parameter. In contrast with [4], the range of working distance analyzed is between 0.25 m and 1 m for the application of visual servoing.

This paper is organized as follows: In Section 2, a visual servoing approach for guiding a robot by using an eye-in-hand ToF camera is presented. Section 3 describes the operation principle of the ToF cameras and the PMD camera employed. In Section 3, an offline camera calibration approach for computing the required integration time from an amplitude analysis is shown. In Section 5, an algorithm for updating the integration time during the visual servoing task is described. In Section 6, experimental results confirm the validity of the visual servoing system and the calibration method. The final Section presents the main conclusions.

## 2. Visual Servoing Using Range Images

**Figure 1.**
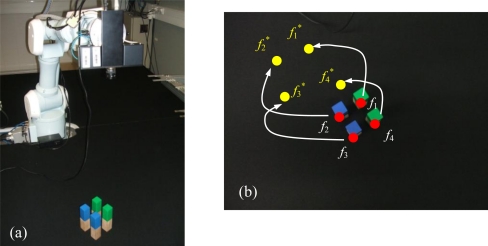
**(**a) Eye-in-hand configuration. (b) Image acquired from the range camera point of view.

A visual servoing task can be described by an image function, **e**_t_, which must be regulated to 0:

(1) $$e_{t}=s-s^{*}$$

where **s** = (*f*_1_, *f*_2,_ … *f*_M_) is a M × 1 vector containing M visual features observed at the current state (*f*_i_ = (*f*_ix_, *f*_iy_)), while $s^{*}=(f_{1}^{*},f_{2}^{*},...f_{M}^{*})$ denotes the visual features values at the desired state, *i.e.,* the image features observed at the desired robot location. In Figure 1(a) the eye-in-hand camera system is shown. A PMD19K camera is located at the end-effector of a 7 d.o.f Mitsubishi PA-10 robot that acquires grayscale images of 160 × 120. In Figure 1(b), an example of a visual servoing task is represented. This figure represents the initial and desired image features from the camera point of view.

**L**_s_ represents the interaction matrix which relates the variations in the image with the variations in the camera pose [1]:

(2) $$\dot{s}=L_{s}\cdot \dot{r}$$

where **ṙ** represents the camera velocity.

By imposing an exponential decrease of **e**_t_ (**ė**_t_ = −*λ*_1_**e**_t_) it is possible to obtain the following control action for a classical image-based visual servoing:

(3) $$v_{c}=-\lambda_{1}{\hat{L}}_{s}^{+}\left(s-s^{*}\right)$$

where *λ*_1_ > 0 is the control gain, ${\hat{L}}_{s}^{+}$ is the pseudoinverse of an approximation of the interaction matrix and **v**_c_ is the eye-in-hand camera velocity obtained from the control law in order to continuously reduce the error **e**_t_. ${\hat{L}}_{s}^{+}$ is chosen as the Moore-Penrose pseudoinverse of **L̂**_s_ [1]. In order to completely define the control action, the value of the interaction matrix for the visual features extracted from the range images will be obtained in the following paragraphs.

First, the interaction matrix will be calculated when only one image feature (*f*_x_, *f*_y_) is extracted. The transformation between the range image **I**(i,j) and 3D coordinates (relative to the camera position) is given by [10]:

(4) $$\begin{array}{c} x_{P}^{C}=z_{P}^{C}\frac{f_{x}^{\prime }}{s_{x}f}\\ y_{P}^{C}=z_{P}^{C}\frac{f_{y}^{\prime }}{s_{y}f}\\ z_{P}^{C}=I\left(f_{x},f_{y}\right)\frac{f}{\sqrt{f^{2}+{\left(\frac{f_{x}^{\prime }}{s_{x}}\right)}^{2}+{\left(\frac{f_{y}^{\prime }}{s_{y}}\right)}^{2}}} \end{array}$$

where f is the camera focal length, s_x_ and s_y_ are the pixel size in the x and y directions and $f_{x}^{\prime }$, $f_{y}^{\prime }$ are the normalized pixel coordinates, relative to the position (u_0_, v_0_) of the optical center on the sensor array $\left(f_{x}^{\prime }=f_{x}-u_{0},f_{y}^{\prime }=f_{y}-v_{0}\right)$.

To obtain the interaction matrix, the intrinsic parameters ξ = (f_u_, f_v_, u_0_, v_0_) are considered, where f_u_ = f·s_x_ and f_v_ = f·s_y_. Therefore, considering these intrinsic parameters, Equation (4) is equal to:

(5) $$\begin{array}{c} x_{P}^{C}=z_{P}^{C}\frac{f_{x}-u_{0}}{f_{u}}\\ y_{P}^{C}=z_{P}^{C}\frac{f_{y}-v_{0}}{f_{v}}\\ z_{P}^{C}=I\left(f_{x},f_{y}\right)\frac{1}{\sqrt{1+{\left(\frac{f_{x}-u_{0}}{f_{u}}\right)}^{2}+{\left(\frac{f_{y}-v_{0}}{f_{v}}\right)}^{2}}} \end{array}$$

From (5) the coordinates of the image feature can be obtained as:

(6) $$\left[\begin{array}{c} f_{x}\\ f_{y} \end{array}\right]=\left[\begin{array}{c} u_{0}\\ v_{0} \end{array}\right]+\frac{1}{z_{P}^{C}}\left[\begin{array}{cc} f_{u} & 0\\ 0 & f_{v} \end{array}\right]\left[\begin{array}{c} x_{P}^{C}\\ y_{P}^{C} \end{array}\right]$$

The time derivative of the previous equation is:

(7) $$\left[\begin{array}{c} {\dot{f}}_{x}\\ {\dot{f}}_{y} \end{array}\right]=-\frac{{\dot{z}}_{P}^{C}}{{\left(z_{P}^{C}\right)}^{2}}\left[\begin{array}{cc} f_{u} & 0\\ 0 & f_{v} \end{array}\right]\left[\begin{array}{c} x_{P}^{C}\\ y_{P}^{C} \end{array}\right]+\frac{1}{z_{P}^{C}}\left[\begin{array}{cc} f_{u} & 0\\ 0 & f_{v} \end{array}\right]\left[\begin{array}{c} {\dot{x}}_{P}^{C}\\ {\dot{y}}_{P}^{C} \end{array}\right]$$

Considering the camera velocity ${\dot{x}}_{P}^{C}$, ${\dot{y}}_{P}^{C}$, ${\dot{z}}_{P}^{C}$ divided in translational ${\dot{x}}_{t}^{C}$, ${\dot{y}}_{t}^{C}$, ${\dot{z}}_{t}^{C}$ and rotational velocity *α̇*^C^, *β̇*^C^, *γ̇*^C^, the following expression can be obtained from Equation (7):

(8) $$\dot{s}=\left[\begin{array}{l} {\dot{f}}_{x}\\ {\dot{f}}_{y} \end{array}\right]=-\frac{-x_{P}^{C}{\dot{\beta }}^{C}+y_{P}^{C}{\dot{\alpha }}^{C}+{\dot{z}}_{t}^{C}}{{\left(z_{P}^{C}\right)}^{2}}\left[\begin{array}{cc} f_{u} & 0\\ 0 & f_{v} \end{array}\right]\left[\begin{array}{l} x_{P}^{C}\\ y_{P}^{C} \end{array}\right]+\frac{1}{z_{P}^{C}}\left[\begin{array}{cc} f_{u} & 0\\ 0 & f_{v} \end{array}\right]\left[\begin{array}{l} -y_{P}^{C}{\dot{\gamma }}^{C}+z_{P}^{C}{\dot{\beta }}^{C}+{\dot{x}}_{t}^{C}\\ -z_{P}^{C}{\dot{\alpha }}^{C}+x_{P}^{C}{\dot{\gamma }}^{C}+{\dot{y}}_{t}^{C} \end{array}\right]$$

Developing the previous equation, an expression which relates the time derivative of the image features with the camera translational and rotational velocity can be obtained:

(9) $$\dot{s}=\underset{L_{s}}{\underset{︸}{\left[\begin{array}{cccccc} \frac{f_{u}}{z_{P}^{C}} & 0 & -\frac{\left(f_{x}-u_{0}\right)}{z_{P}^{C}} & -\frac{\left(f_{x}-u_{0}\right)\left(f_{y}-v_{0}\right)}{f_{v}} & \frac{{\left(f_{x}-u_{0}\right)}^{2}+f_{u}^{2}}{f_{u}} & -\frac{f_{u}\left(f_{y}-v_{0}\right)}{f_{v}}\\ 0 & \;\frac{f}{z_{P}^{C}} & -\;\frac{\left(f_{y}-v_{0}\right)}{z_{P}^{C}} & -\frac{{\left(f_{y}-v_{0}\right)}^{2}-f_{v}^{2}}{f_{v}} & \frac{\left(f_{x}-u_{0})(f_{y}-v_{0}\right)}{f_{u}} & \frac{f_{v}(f_{x}-u_{0})}{f_{u}} \end{array}\right]}}.\left[\begin{array}{l} {\dot{x}}_{t}^{C}\\ {\dot{y}}_{t}^{C}\\ {\dot{z}}_{t}^{C}\\ {\dot{\alpha }}^{C}\\ {\dot{\beta }}^{C}\\ {\dot{\gamma }}^{C} \end{array}\right]$$

where:

(10) $$z_{P}^{C}=I\left(f_{x},f_{y}\right)\frac{1}{\sqrt{1+{\left(\frac{f_{x}-u_{0}}{f_{u}}\right)}^{2}+{\left(\frac{f_{y}-v_{0}}{f_{v}}\right)}^{2}}}$$

The matrix obtained in Equation (9) is the interaction matrix, **L**_s_, therefore, **s⋅** = **L**_s_ · **ṙ**. The pseudo inverse of the interaction matrix derived in (9) is calculated for the control action in (3). In this last equation, an approximation of the interaction matrix is considered due the necessity of estimating the camera intrinsic parameters, ξ. If M visual features can be extracted from the image, the interaction matrix can be obtained as **L**_s_= [**L**_s1_ **L**_s2 …_ **L**_sM_]^T^, where **L**_si_ is the interaction matrix determined in (9) for only one feature.

Various previous works have studied the image-based visual servoing stability. In applications with commercial robots the complete dynamical robot model is not provided. In this cases, the system stability is deduced depending on kinematics properties [11–14]. Paper [1] describes that the local asymptotic stability can be ensured when the number of rows of the interaction matrix is greater than 6. However, we cannot ensure global asymptotic stability. As is indicated in [1], to ensure the local stability, the desired visual features must be closed the current ones. Furthermore, ${\hat{L}}_{s}^{+}$ and $L_{s}^{+}$ must be equal or very similar. To do so, the camera depth and intrinsic parameters must be correctly computed. The following algorithm [15] has been used in order to estimate the camera intrinsic parameters. In addition, the accurate determination of the camera depth is one of the main problems. It will be solved in the following sections.

## 3. Analysis of the Distance Measurement Computed with the ToF Camera

In this section, a behaviour analysis of ToF cameras is provided. This analysis helps to define the methods to improve the depth measurement which will be used in the visual servoing system. A PMD19K camera has been used in this analysis. The PMD19K camera contains a Photo Mixer Device (PMD) array with a size of 160 × 120 pixels. This technology is based on CMOS technology and a time-of-flight (ToF) principle.

There are other similar cameras based on the same principle and with CMOS technology such as the CamCube 2 or 3 of PMD-Technologies and the SR2, SR3000 or SR4000 of CSEM-Technologies. The specifications and a comparison of the behaviour of these cameras is available in [8] and [16], respectively. PMD19K works with a wavelength of near-infrared (NIR) light of 870 nm and it can capture up to 15 fps with a depth resolution of 6 mm. Furthermore, in the experiments here presented, the camera is connected by Ethernet and it is programmable by SDK for Windows, although it can be connected by Firewire interface and programmable for Linux, too. The ToF camera technology is based on the principle of modulation interferometry [6,16]. The scene is illuminated with NIR light (PMD19K module with a default frequency of ω = 20 Mhz) and this light is reflected by the objects in the scene. The difference between both signals, emitted and reflected, causes a phase delay which is detected for each pixel and used to estimate the distance value. Thus, the ToF camera provides $2\frac{1}{2}$ D depth information of dynamic or static scenes irrespective of the object’s features such as: intensity, depth and amplitude data simultaneously for each pixel of each image captured. The intensity represents the grayscale information, the depth is the distance value calculated within the camera and the amplitude is the signal strength of the reflected signal (quality of depth measures). Then, given the speed light, c, the frequency modulation, ω, the correlation between signals for four internal phase delays, r_0_(0°), r_1_(90°), r_2_(180°), r_3_(270°), the camera compute the phase delay, ϕ, the amplitude, a, and the distance between sensor and the target, z, as follows:

(11) $$\phi =\text{arctan}\left(\frac{r_{1}-r_{3}}{r_{0}-r_{2}}\right)$$

(12) $$a=\frac{\sqrt{{\left(r_{1}-r_{3}\right)}^{2}+{\left(r_{0}-r_{2}\right)}^{2}}}{2}$$

(13) $$z_{P}^{C}=\frac{c\phi }{4\pi \omega }$$

This type of cameras has some disadvantages [17]: they are sensitive to background light and interferences and they cause oversaturation and underexposure pixels. The PMD camera has two adjustable parameters to attenuate these errors in the pixels: the modulation frequency and the integration time. To do not change the original calibration determined by the manufacturer, only the behaviour of integration time has been studied to be adjusted. The integration time is defined as the exposure time or the effective length of time a camera’s shutter is open. This is time is needed so that the light reachs the image sensor suitably.

In a visual servoing system with eye-in-hand configuration (Figure 1) the camera is mounted at the end-effector of a robotic arm. Therefore, when the robot is moved, the distance between sensor and target, $z_{P}^{C}$, changes and the integration time, τ, has to be on-line adjusted to minimize the error in the computed depth. Whenever this parameter is suitably computed, the image range can be acquired in better conditions and so the features extraction process in the image can be improved looking for reaching the best features without modified the light environment or the object surfaces in the scene.

**Figure 2.**
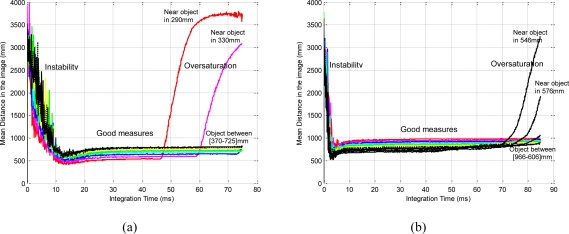
Evolution of the mean distance of the range image for two different scenes: (a) An object and the camera moved between 0.5 m and 1 m. (b) Four objects and the camera moved between 0.3 m and 0.8 m.

Figure 2 shows the stability of the distance measurements obtained from the range images with regards to the integration time. May *et al.* [9] show this dependency in a Swisrange SR-2 camera for the navigation of a mobile robot. The same is studied by Wiedemman *et al.* [8] to build maps with a mobile robot and by Gil *et al.* [17] to guide a robotic arm by using an eye-in-hand configuration for visual servoing (Figure 1). In this last work, a PMD19K camera was used.

In previous works, some experiments were done in order to observe the evolution of the distance measured by the camera when the integration time changed. In those experiments from 750 images (an integration time offset of 100 ms between each image), a relationship between mean distance value, $z_{P}^{C}$, and integration time, τ, in microseconds is shown when the robot (Figure 1) is moved and the distance between sensor and target changes. As Figure 2 shows, when integration time is small, the distance computed is unstable and nontrustworthy. In the same way, when the integration time is high an oversaturation phenomenon sometimes appears in the signal which determines the distance curves. Normally, this phenomenon only appears when the distance measured between scene and camera is below a fixed nominal distance or distance threshold, as it is explained in [17]. In Figure 2(a), oversaturation appears when the integration time is greater than 45 ms. However, in Figure 2(b), the oversaturation only occurs when the integration time is greater than 70 ms. Therefore, the nearer the target is, the smaller the threshold of integration time must be. Thus, the farther the target is, the more precise the distance computed is. In addition, something similar happens with the intensity as it is explained in [9], although it is more sensitive to the background light and interferences [8,12]. Consequently, in the calibration process, the flat zone of the curve (Figure 2) has to be computed in order to use a ToF camera such as PMD19K for visual servoing. This zone determines the minimum and maximum integration times allowed to avoid the oversaturation and the instability problems. In this paper, these values have been fixed using the calibration method presented in [17], where the histogram which represents the frequency distributions of the amplitude measurements of PMD19k are adjusted by means of probability density functions (PDF) using Kolmogorov-Smirnov and Anderson-Darling methods.

## 4. Camera Calibration: Computing Integration Time from an Amplitude Analysis

**Figure 3.**
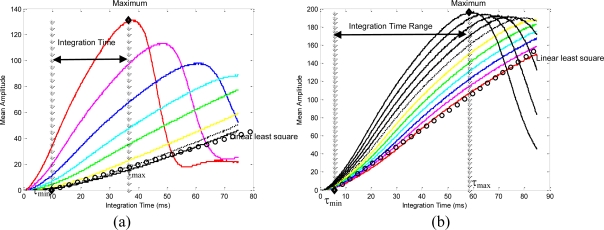
Evolution of mean amplitude, a_m_, for the tests of Figure 2.

As regards the amplitude measurements, the curve which shows the evolution of the mean amplitude can be computed from a set of images acquired using a nominal fixed distance (the same as the mean distance that was computed in Figure 2). The analysis of the mean amplitude curve determines the thresholds of time integration, [τ_min_, τ_max_] which are needed in order to guarantee the precise computation of the distance measurements (Figure 3). The amplitude parameter, a, of a ToF camera defines the quality of the range images computed using a specific integration time. The minimum threshold, τ_min_, is computed as the minimum integration time needed to compute the image depth in the desired camera location. It is determined as the time value where a least squares line fitting the mean amplitude curve crosses the zero axis (Figure 3). The maximum threshold, τ_max_, is computed as the maximum integration time needed to compute the image depth in the initial camera location. These limits (Figure 3) are computed depending on the distance between target and camera by means of an offline process, as follows:

Pose the Robot in the initial pose and capture an image, I*_τ_*, for some integration time, *τ* ∈ [0,85ms] At each iteration:

1. Compute mean amplitude: a_m_
2. Estimate the frequency histogram for a_m_ and fit it by means of K-S and A-D Tests in order to classify the scene according to look-up-table as near or far target
  - τ_min_ is computed from zero crossing determinated by the fitting of the curve which represents the image at the maximum distance (min{*τ*} to capture the image at maximum working distance) (see Figure 3)
  - τ_max_ is computed as the suitable integration time for obtaining a desired mean amplitude, a_d_, such as:
    $$\text{If}\;(\text{near})\;\text{then}\;a_{d}\;=\;\text{max}\{a_{m}\}\;\text{else}\;a_{d}=\text{upper}\_\text{quartile}\{a_{m}\}$$

The amplitude analysis of Figure 3 shows a group of curves (a curve for each camera location). The curves show how the linearisation level (part of flat slope) determines the degree of oversaturation. Thus, the amplitude curves grow quickly until they reach an absolute maximum value when the camera is near the target and the curves are more linear when the camera is moved away from the target.

Once, the integration time values for final and initial camera positions have been computed, some intermediate integration time, τ_k_, Figure 3(a) are computed for the robot trajectory. To do this, empirical tests have been done with the following algorithm:

1. Fix the integration time as *τ*_0_ = *τ*_max_ for image I_0_
2. Compute the deviation error e_a_ = a_d_ – (a_m_)_0_ where a_d_ = max{a_m_} according to a desired minimum distance.
3. Update integration time following the control law *τ*_k_ = *τ*_k–1_ (1 + K · e_a_) where K is a proportional constant and it is adjusted depending on the robot velocity.

**Figure 4.**
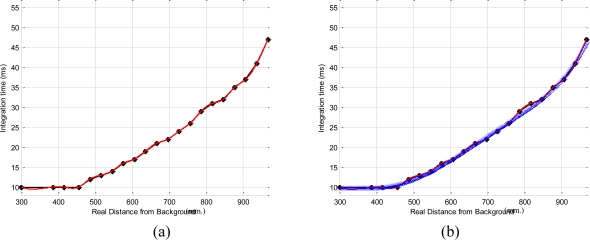
Polynomial interpolation applied to compute $\frac{\partial \tau }{{\partial z}_{P}^{C}}$ for distances between 0.3 and 1 m, for the tests of Figure 2.

This way, some intermediate integration time values, *τ*_k_ ∈ [*τ*_min_, *τ*_max_], have been estimated for different distances between the final and the initial positions. Therefore, the proper computation of $\frac{\partial \tau }{\partial z_{P}^{C}}$ is done using a polynomial interpolation which fits these intermediate positions (Figure 4). In general, polynomial interpolation may not fit precisely at the end points. But this is not a problem because they are fixed with the time integration needed for the desired and the initial camera positions. Considering, τ_min_ and τ_max_ as the values 10 ms and 46.4 ms (upper quartile of the maximum value shown in Figure 3(b), 57.4 ms.) respectively and some intermediate time, τ_k_, all computed, according the previous calibration method, $\frac{\partial \tau }{{\partial z}_{P}^{C}}$ is given by:

(14) $$\frac{\partial \tau }{{\partial z}_{P}^{C}}=2.8825z_{\mu }^{4}+4.5556z_{\mu }^{3}-4.581z_{\mu }^{2}+0.4968z_{\mu }+11.8853$$

where:

(15) $$z_{\mu }=\frac{z_{P}^{C}-662}{193}$$

## 5. Algorithm for Updating the Camera Integration Time During the Task

From the previous analysis, a method to automatically update the integration time is presented in this section in order to be applied during visual servoing tasks.

Considering ^c^**M**_o_ the extrinsic parameters (pose of the object frame with respect to the camera frame), an object point can be expressed in the camera coordinate frame as:

(16) $$P_{P}^{C}\left(x_{P}^{C},y_{P}^{C},z_{P}^{C}\right)=M_{O}^{C}P_{P}^{O}$$

Considering a pin-hole camera projection model, the point $P_{P}^{C}$ with 3D coordinates relative to the camera reference frame is projected onto the image plane at the point **p** of 2D coordinates. This point is computed from the focal length (distance between retinal plane and optical center of camera) as:

(17) $$p={\left(x,y\right)}^{T}={\left(f\frac{x_{P}^{C}}{z_{p}^{C}},f\frac{y_{P}^{C}}{z_{P}^{C}}\right)}^{T}$$

Finally, the units of (17) specified in terms of metric units (e.g., mm.) are scaled and transformed in coordinates in pixels relative to the image reference frame, as:

(18) $$s=\left(f_{x},f_{y}\right)=\left(u_{0}+f_{u}x,v_{0}+f_{v}y\right)$$

where ξ = (f_u_, f_v_, u_0_, v_0_) are the camera intrinsic parameters.

The intrinsic parameters describe properties of the camera used, such as the position of the optical center (u_0_, v_0_), the size of the pixel and the focal length defined by (f_u_, f_v_). They are computed from a calibration process based on [15]

During a visual servoing task, the camera extrinsic parameters are not known, and ^c^**M**_o_ is considered as an estimation of the real camera pose. In order to determine this pose, we must minimize progressively the error between the observed data, **s**_o_, and the position of the same features computed by back-propagation employing the current extrinsic parameters, **s** (16)–(18). Therefore an error function which must be progressively reduced is defined as:

(19) $$e=s-s_{o}$$

The time derivative of **e** will be:

(20) $$\dot{e}=\dot{s}-{\dot{s}}_{o}=\frac{\partial s}{\partial r}\frac{\partial r}{\partial t}=L_{s}\frac{\partial r}{\partial t}$$

To make **e** decrease exponentially to 0, **ė** = −λ_2_**e**, we obtain the following control action:

(21) $$\frac{\partial r}{\partial t}=-\lambda_{2}L_{s}^{+}e$$

where λ_2_ is a positive control gain and $L_{s}^{+}$ is the pseudoinverse of the interaction matrix (9). Once the error is annulled the extrinsic parameters will be obtained. This approach is used by the virtual visual servoing systems to compute the camera locations. More details about the convergence, robustness and system stability can be seen in [11,12].

Consequently, two estimations are obtained for the depth of a given image feature: one depth (*z*_1_) from the previous estimated extrinsic parameters and another depth ( $z_{2}=z_{P}^{C}$) from (10). This last depth is calculated from the range image and, therefore, can be updated by modifying the camera integration time. The adequate integration time will be obtained when *z*_1_ and *z*_2_ are equal. Therefore, a new control law is applied in order to update the integration time, τ, by minimizing the error between *z*_1_ and *z*_2_:

(22) $$\frac{\partial \tau }{\partial t}=-\lambda_{3}\frac{\partial \tau }{{\partial z}_{p}^{C}}(z_{2}-z_{1})$$

where λ_3_ > 0.

The algorithm for updating the camera integration time is summarized in the following lines: First perform the offline camera calibration to determine the initial integration time and $\frac{\partial \tau }{{\partial z}_{P}^{C}}$ (see Section 4).

At each iteration of the visual servoing task:

1. Apply the control action to the robot: $v_{c}=-\lambda_{1}{\hat{L}}_{s}^{+}\left(s-s^{*}\right)$
2. Estimate the extrinsic parameters using virtual visual servoing.
3. Determine the depth, *z*_1_, from the previous extrinsic parameters and *z*_2_ from the range image (10).
4. Update the integration time by applying $\frac{\partial \tau }{\partial t}=-\lambda_{3}\frac{\partial \tau }{\partial z_{p}^{C}}\left(z_{2}-z_{1}\right)$

**Figure 5.**
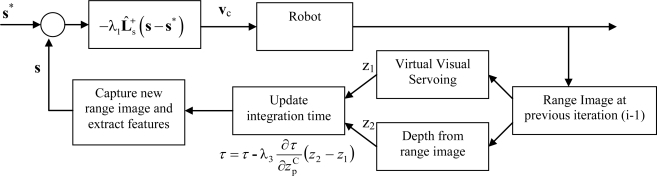
Block diagram of the visual servoing system.

In order to describe more clearly the interactions among all the subsystems that compose the proposed visual servoing system, a block diagram is represented in Figure 5. In this block diagram (Figure 5) it is possible to observe that in the feedback of the visual servoing system a complete convergence of virtual visual servoing is performed in order to determine the extrinsic parameters. Moreover, the convergence and stability aspects when virtual visual servoing techniques are used as feedback of a visual servoing system are discussed in [18].

## 6. Results

The target used for the experiments can be seen in Figure 1. This target is composed of four objects on a black table as background to ensure a low reflectivity at the borders. The PMD19k is mounted at the end-effector of a Mitsubishi PA10 with 7 d.o.f. In addition, the ambient light (exterior light source) was controlled with a power regulator for this work in indoor environments. Thereby, special care was taken to avoid the interference with the NIR of the camera.

### 6.1. Trajectory 1

**Figure 6.**
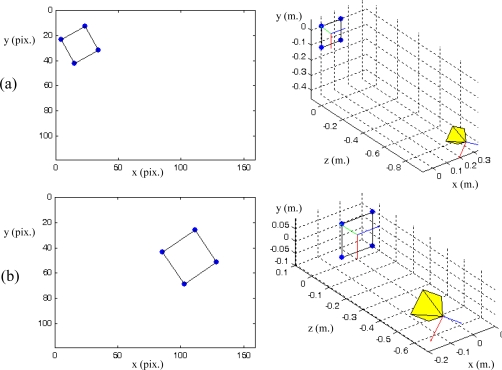
(a) Initial position of the image features and the eye-in-hand camera. (b) Final position of the image features and the eye-in-hand camera. (Trajectory 1).

The real distance between camera and target (background and objects) for this first experiment was $600<\;z_{P}^{C}\;<966\;\text{mm}$. The initial and final camera locations were $P_{\text{Pi}}^{C}=\left(0,0,966\right)\text{mm}$ and $P_{\text{Pf}}^{C}=\left(-100,-200,600\right)\text{mm}$ respectively. The features are computed as the centroid of the four objects represented in the range image acquired by the PMD19k (Figure 1). The pixel coordinates of these centroids are **p_1_** = (*7,23*)^T^, **p_2_** = (*27,12*)^T^, **p_3_** = (*17,41*)^T^ and **p_4_** = (*37,29*)^T^ for the initial robot pose and **p_1_** = (*85,40*)^T^, **p_2_** = (*115,24*)^T^, **p_3_** = (*103,71*)^T^ and **p_4_** = (*134,52*)^T^ for the final pose. Figure 6 depicts the initial and final positions of the visual features and the eye-in-hand camera.

**Figure 7.**
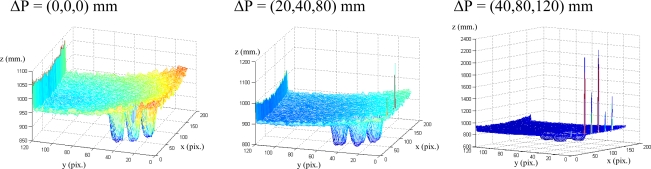
Range Image computed for the integration time of 53 ms.

In Figure 7, the measured depth data from a range image is shown for three different camera locations. Only a range image was plotted but from three different camera location (offset Δ**P** = (Δ*X*, Δ*Y*, Δ*Z*)mm between locations) with the same time integration value, 53 ms. This plot shows distinct systematic errors when the integration time is not updated or it is chosen nadequately. However, these errors can be easily corrected by applying the method presented in Section 5. Thus, the combination of the calibration method for estimating the integration time in the initial position [17] and the method to update the integration time presented in sections 4 and 5 significantly improves the quality of the measured depth data.

**Figure 8.**
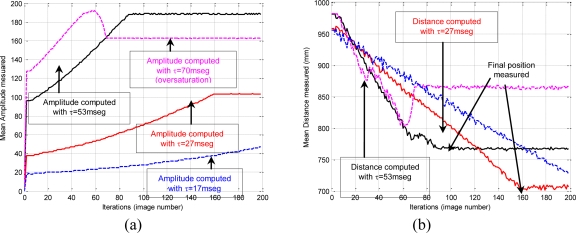
(a) Evolution of the measured amplitude when the integration time is not updated. (b) Evolution of the depth parameter when the integration time is not update.

Furthermore, Figure 8 shows how the depth and amplitude measured by the PMD19k change when the integration time is not updated to adapt it according to the distance between camera and target when the robot is moving. The PMD19K has been configured with some different integration times (17, 27, 53 and 70 ms). For example, 53 ms and 27 ms are near the good integration times for the initial and final camera locations, respectively. The experimental results show that whenever an integration time is greater than the optimal value (such as 70 ms), the amplitude values show instability after the maximum amplitude is reached (Figure 8(a)). Furthermore, if the used integration time is smaller than the optimal value (such as 17 ms), so many iterations are needed until the distance is computed correctly (Figure 8(b)). However, the time 27 ms compute a depth for the final position close to the final camera location.

**Figure 9.**
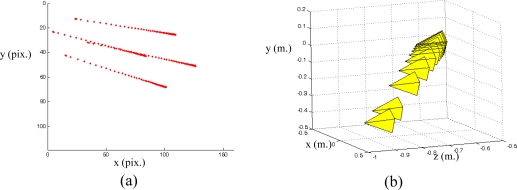
Trajectory during the visual servoing task. (a) Trajectory of the image features. (b) Trajectory of the eye-in-hand camera. Experiment 1.

**Figure 10.**
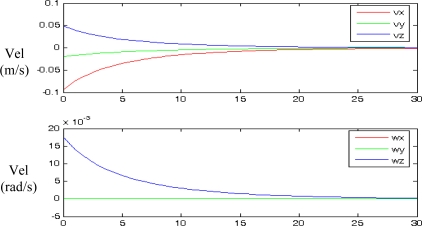
Velocities during the visual servoing task. Experiment 1.

Applying the algorithm described in Section 5 from the initial and desired image features location, the image trajectory presented in Figure 9(a) is obtained. In this figure, it is possible to observe that the image features follow a straight line between the initial and the final locations. Furthermore, in Figure 9(b), the camera poses during the visual servoing task are represented. It is possible to observe that the visual servoing task is correctly performed. Therefore, we can conclude that a correct behaviour is obtained in the image and in the 3D space. In Figure 10 the velocities of the robot’s end-effector applied during the visual servoing task are represented.

**Figure 11.**
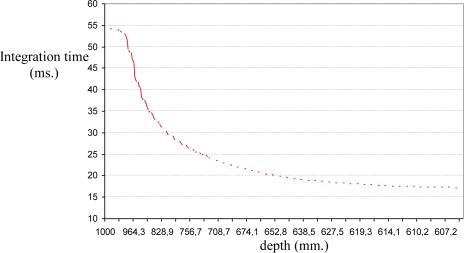
Integration time values at each iteration of the visual servoing task. Trajectory 1.

**Figure 12.**
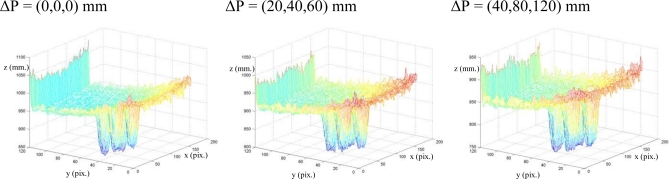
Range Image computed for the integration time updated at each iteration.

In order to perform the correct tracking, the integration time is updated at each iteration of the visual servoing task using the algorithm described in Section 5. Figure 11 shows the value of the integration time considered at each iteration. Finally, considering these values of the integration time, the new range images obtained at ΔP = (0,0,0) mm, ΔP = (20,40,80) mm and ΔP = (40,80,120) mm are represented in Figure 12. Comparing these figures with the ones obtained at Figure 7, it is possible to observe that the update process of the integration time based on the proposed algorithm eliminates the the previous errors.

The image ranges shown in Figure 11 are better than those in Figure 6 because the integration time has been updated during the visual servoing task. The distance between the camera and the target has changed as Figure 10 shows and the camera PMD19k has been self-configured with suitable integration time values. In this example, the integration times have been (53, 41 and 35 mseg).

### 6.2. Trajectory 2

**Figure 13.**
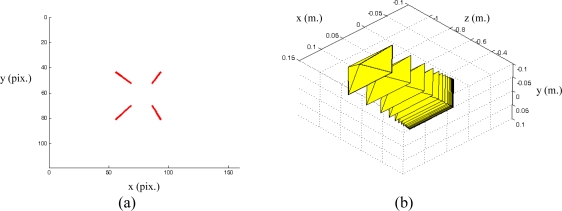
Trajectory during the visual servoing task. (a) Trajectory of the image features. (b) Trajectory of the eye-in-hand camera. Experiment 2.

**Figure 14.**
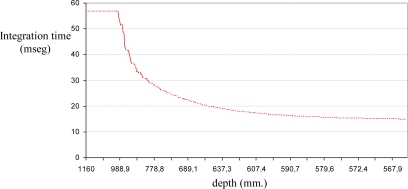
Integration time values at each iteration of the visual servoing task. Experiment 2.

In this case, a trajectory with a displacement only in depth is described. The initial and final positions of the features in the image are (68,51)(86,51)(68,70)(86,70) and (56,43)(93,43)(56,80) (93,80), respectively. The initial distance between the eye-in-hand camera and the object is 1,160 mm and the final distance is 560 mm by using the proposed control law, the robot is able to perform precisely the displacement in depth as Figure 13 shows. In order to complete the task, the integration time has been updated using the algorithm described in Section 5 and thus the evolution represented in Figure 14 is obtained. As we have previously indicated [see Figure 2(b)], the minimum, τ_min_, and maximum,τ_max_, values of the integration time are 10 ms and 57.4 ms, respectively. Therefore, when the theoretical value for the integration time is greater than τ_max_ this parameter is saturated to 57.4 ms (see Figure 14).

### 6.3. Trajectory 3

**Figure 15.**
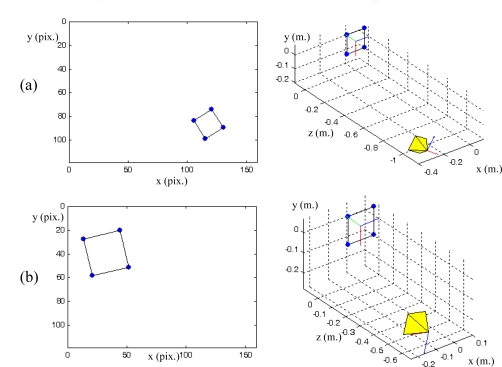
(a) Initial position of the image features and the eye-in-hand camera. (b) Final position of the image features and the eye-in-hand camera. Experiment 3.

As described in [1], in classical image-based visual servoing systems the depth of each image feature must be estimated at each iteration of the control scheme. In order to avoid the necessity of estimating these parameters, one popular approach is to choose ${\hat{L}}_{s}^{+}=L_{s^{*}}^{+}$, where **L**_s^*^_ is the value of **L**_s_ for the desired position s^*^. In this case, $L_{s^{*}}^{+}$ is constant, an only the desired depth of each point has to be set, and thus, no varying 3D parameters have to be estimated during the visual servoing. In this section, a comparison between this last approach and the one proposed in this article is shown. To do so, a visual servo task is considered in which the initial position of the visual features in the image are (105,83)(119,73)(114,98)(130,89) and the desired position for the image features are (13,27)(44,20) (20,58)(51,51) [Figure 15(a)]. The initial and final positions of the eye-in-hand camera are represented in Figure 15(b).

**Figure 16.**
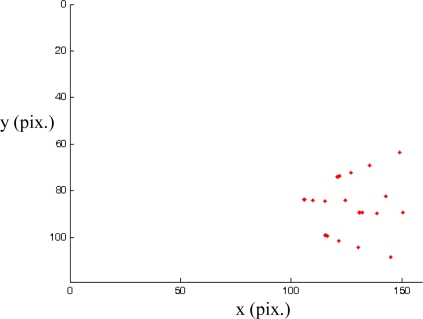
Image trajectory when ${\hat{L}}_{s}^{+}=L_{s^{*}}^{+}$.

**Figure 17.**
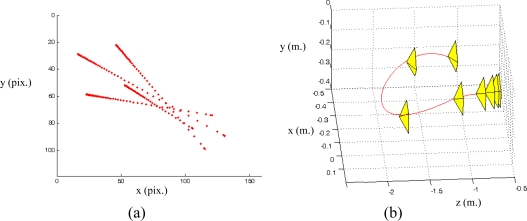
Trajectory during the visual servoing task. (a) Trajectory of the image features. (b) Trajectory of the eye-in-hand camera. Experiment 3.

Figure 16 shows the evolution of the image features which are obtained when a classical image-based visual servoing system with ${\hat{L}}_{s}^{+}=L_{s^{*}}^{+}$ is applied. In this case, the visual features are lost and the image features does not converge towards the desired ones. However, the use of the control law and the depth estimation proposed in Equations (9) and (10) generates the behaviour represented in Figure 17. In this last figure we can see that the visual servoing system is able to converge towards the desired location. This experiment shows the necessity of correctly estimating the depth parameters in order assure the correct convergence.

**Figure 18.**
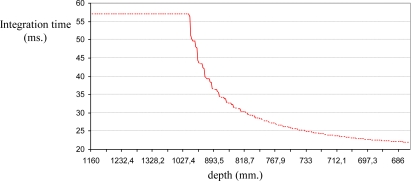
Integration time values at each iteration of the visual servoing task. Experiment 3.

In this experiment there are important variations in the distance between the camera and the object from which the features are extracted. The initial and final depths are 1,160 mm. and 680 mm. respectively, and during the task the depth arrive until 1,760 mm. Thus, considering a fixed integration time, important errors appear and the task cannot be performed. Therefore, the integration time has to be updated with the approach described in this paper, and thus the evolution represented in Figure 18 is obtained. In this experiment the integration time is limited to values between the minimum, τ_min_, and maximum, τ_max_, in the same way that in the previous experiment according to Figure 2(b).

## 7. Conclusions

This paper presents a new image-based visual servoing system which integrates range information in the interaction matrix. Another property of the proposed system is the possibility of performing the camera calibration during the task. To do this, the visual servoing system uses the range images not only to determine the depths of the object features but also to adjust the camera integration time during the task.

When a ToF camera is employed to guide a robot, the distance between the camera and the objects of the workspace change. Therefore, the camera integration time must be updated in order to correctly observe the objects of the workspace. As it is demonstrated in the experiments, the integration time must be updated depending on the distance between the camera and the objects. The use of the proposed approach guarantees that the information obtained from the ToF camera is accurate because an adequate integration time is employed at each moment. This last aspect permits obtaining a better estimation for the objects depth. Therefore, the behaviour of the visual servoing is enhanced with respect to previous approaches where this parameter is not accurately estimated. Currently, we are working in determining the accurate dynamic model of the robot to improve the visual servoing control law in order to assure the given specifications during the task.
